# Survey of Korean Medicine Doctors’ Practices in Treating Shoulder Pain: A Cross-Sectional Study

**DOI:** 10.3390/healthcare13131482

**Published:** 2025-06-20

**Authors:** Jae-Won Park, Yunkyung Song, Dong-Ho Keum, Seo-Hyun Park

**Affiliations:** 1 College of Korean Medicine, Dongguk University Graduate School, Seoul 04620, Republic of Korea; jaewon012@dongguk.edu; 2 Department of Korean Rehabilitation Medicine, Gachon University College of Korean Medicine, 21 Keunumul-ro, Jung-gu, Incheon 22318, Republic of Korea; lyricsong@gachon.ac.kr; 3 Department of Rehabilitation Medicine of Korean Medicine, Dongguk University Bundang Oriental Hospital, Seongnam-si 13601, Republic of Korea; 19950027@gwmail.dongguk.edu

**Keywords:** shoulder pain, CPG, national survey, Korean medicine

## Abstract

**Background:** Shoulder pain affects 20–40% of the global population and is associated with substantial healthcare costs. This study aimed to investigate the current clinical practice for shoulder pain in Korean medicine (KM) clinics and collate the insights to suggest updates to the Korean Clinical Practice Guidelines (KCPGs) for shoulder pain. **Methods:** A web-based survey was conducted among Korean Medicine Doctors (KMDs) from 6 March 2023 to 27 March 2023. The survey comprised 28 questions related to clinical practice, diagnosis, treatment, progress, prognosis, perception of KM on shoulder pain, and demographic characteristics. Some questions accepted multiple responses, whereas some were scaled response questions. Statistical analyses were performed on the collected data. **Results:** In total, 788 KMDs participated in the survey. More than 40% reported treating over 20 shoulder pain patients monthly. Diagnosis primarily relied on pain pattern, range of motion (ROM), activities of daily living (ADL), and social history. Prognostic valuation also relied on these parameters. Most KMDs used multiple treatment methods, including acupuncture, pharmacopuncture, cupping therapy, Chuna therapy, and herbal medicine. **Conclusions:** KMDs adopted a comprehensive multifactorial approach toward shoulder pain treatment. Insights from this survey will help update the previous KCPG for shoulder pain.

## 1. Background

Shoulder pain is characterized by pain associated with tissues surrounding the shoulder joint [1]. Its main symptoms are ‘pain in the shoulder joint’ and ‘limitation of the range of motion (ROM),’ which result from degenerative changes in the shoulder joint, ligament damage, muscle rupture, and inflammation of the synovial cyst, and so on.

Shoulder pain is a common problem affecting 20–40% of the global population at least once in their lifetime. In 2022, shoulder pain was ranked ninth in the number of affected outpatients in Korean medicine (KM) clinics [2]. Shoulder pain could be caused by various diseases: frozen shoulder, rotator cuff syndrome, biceps tendinitis, calcific tendinitis of the shoulder, impingement syndrome of the shoulder, and sprains and strains of the shoulder joint [2,3]. Other diseases that do not originate from tissues surrounding the shoulder region can also result in shoulder pain: the lung, heart, and cervical spine. Some specific conditions, such as post-stroke shoulder pain and post-operative shoulder pain, have also been reported [2,3].

As the number of patients with shoulder pain and the total medical insurance costs steadily increase, standardized treatment protocols are needed for efficient treatment, prevention, and daily care [3]. Based on this consideration, various clinical practice guidelines (CPG) have been developed worldwide [4,5,6,7].

The Korean Medicine Clinical Practice Guidelines for shoulder pain (KCPG-shoulder pain) were first published under the supervision of the Korean Institute of Oriental Medicine (KIOM) in 2015 [8], and then updated in 2020 [1]. In recent times, the importance of considering the views of users such as doctors and patients in developing CPG is growing [9]. However, there is a dearth of studies reflecting current trends of KM that contribute to KCPG-shoulder pain. A survey among KM doctors (KMDs) has not yet been conducted with regard to the current trends in KM for treating shoulder pain. Prior attempts to investigate the clinical ground reality of shoulder pain treatment were based on previous studies on KM, surveys of medical doctors (MD) who were not KMDs, or other clinical databases including the national survey for Korean health insurance, with data being collected from individual KM hospitals and clinics [10,11,12]. Given consistent changes in the medical environment, including demographic characteristics of patients and KMDs, common diseases that can cause shoulder pain, and various types of treatments, it is necessary to provide an update on the recent clinical practice status. All reasonable diseases that can result in shoulder pain were included in this survey. However, some diseases not related to tissues surrounding the shoulder region were excluded from the scope of this study. Furthermore, some specific conditions, such as post-stroke shoulder pain and post-operative shoulder pain, were also not reported in this study.

This survey-based study aimed to investigate the current clinical practices among KMDs in treating shoulder pain, with the goal of providing foundational insights to inform future updates to the KCPG-shoulder pain. Specifically, this study seeks to examine how various treatment modalities—such as acupuncture, pharmacopuncture, cupping, and Chuna therapy—are currently utilized in practice and how closely these practices align with the existing KCPG recommendations. We hypothesize that these treatments are often applied in combination and may partially diverge from standardized protocols. By identifying actual usage patterns and areas of discrepancy, this study aims to highlight opportunities for refining the guidelines to better reflect real-world clinical practice and support optimal care.

## 2. Methods

### 2.1. Study Design

This cross-sectional study was designed according to the STORBE (Strengthening the Reporting of Observational Studies in Epidemiology) guideline to investigate the clinical environment of shoulder pain, focusing on understanding the types of treatments used in clinical practice through a self-developed web-based survey of KMDs. The survey questionnaire was developed by three KM specialists of rehabilitation medicine after reviewing previous online national survey-based studies [13,14,15]. The survey questionnaire was developed based on the recommendations and clinical interventions outlined in the previously established KCPG for shoulder pain, aiming to assess the current clinical implementation and perception of those recommendations. To ensure the content validity and reliability of responses, question formats and Likert scale usage were designed with reference to prior national survey studies employing similar methodologies. The draft survey was also reviewed and revised based on expert consultation from Korean medicine specialists. The questionnaire consisted of 28 questions classified into six sections: (1) clinical practice status, (2) diagnosis, (3) treatment (overall and individual KM treatment), (4) progress and prognosis, (5) perception of KM on shoulder pain, and (6) demographic characteristics. Some questions accepted multiple responses. To ensure objectivity in gathering insights regarding the clinical practices and the importance or frequency of each treatment, a 5- or 7-point Likert scale was used for relevant questions.

### 2.2. Participants and Procedures

The web-based survey form was conducted for three weeks from 6 March 2023 to 27 March 2023. An e-mail containing the URL to the survey form was sent to approximately 27,000 KMDs listed as members of the Korean Medical Association. The online survey platform, “Monkey survey”, was used to create the survey form and collect the responses.

### 2.3. Ethics

This study was approved by the Institutional Review Board of Dongguk University Bundang Oriental Hospital (IRB No. DUBOH 2023-0001. Approval date: 1 April 2023). The survey was conducted after obtaining consent for collecting and using personal information and consent for participation in research. All participants were informed about the purpose of the study and the time required for the survey. They were also informed that their personal information would be protected, the survey results would be confidential, and the survey results would be used for academic purposes only. The form also clarified that the survey could be stopped at any time during the survey, and the KMDs also had the option of not entering their personal information.

### 2.4. Statistical Analysis

All responses were analyzed based on the section where they appeared on the survey questionnaire. The responses to each question were organized as numbers and percentages. If it is possible and needed, all continuous variable was calculated and presented as averages. Responses to scale-based questions related to the importance and frequency of diagnosis, progress and prognosis, and KM treatment were collected using a five or seven-point scale.

Those five or seven-point scales were calculated to the average scores using the weighted average by assigning a numerical value to each response option. The lowest response, such as ‘never’, was assigned a value of 1, while higher responses, like ‘sometimes’, ‘often’, or ‘always’, were assigned progressively higher values, with the highest response receiving a value of 5 (or 7, depending on the scale). For each response option, the number of responses was multiplied by the corresponding numerical value. After that, all the results from these multiplications were added up to obtain a total weighted score. Those total scores were divided by the overall number of responses to arrive at the weighted average.

## 3. Results

### 3.1. General Characteristics

In total, 788 (585 male and 203 female) KMDs participated in the survey. In relation to the period of clinical experience, the proportion of responses of ‘6 to 10 years’, ‘11 to 15 years’, and ‘20 to 30 years’ were similarly presented from 20% to 24%. Most participants worked in KM clinics (70.3%). Among all respondents, about half had a Bachelor’s degree (46.2%), and others were more educated. About one fourth (23.6%) had both a Master’s or Ph.D degree and specialization subject in KM. Others had either a Master’s or Ph.D (20.6%) or specialization subject in KM (9.6%). Among the KM specialist, their specialized subject were ranked in ‘internal medicine (18.2%)’, ‘department of acupuncture and moxibustion (17.4%)’, and ‘rehabilitation medicine (13.7%)’ (Table 1).

### 3.2. Clinical Practice Status

On average, more than one-third (n = 332, 42.1%) of KMDs saw more than 20 first-visit patients with shoulder pain in KM institutions per month. The three most common causative diseases for shoulder pain were ‘frozen shoulder (n = 566, 24.8%)’, ‘sprain and strain (n = 523, 22.9%)’, and ‘rotator cuff tear or rupture (n = 450, 19.7%)’. The majority of patients’ ages were presented from 41 to 60 years old (62.4%). The average duration of treatment varied, but ‘1–2 months’ was the most frequent response (n = 303, 38.5%). Treatment satisfaction was concentrated on 7 or 8 points out of 10 [measured by KMDs; 10 indicates 100% satisfaction with treatment; 7, n = 228 (36.5%); 8, n = 271 (34.4%)].

### 3.3. Diagnosis

The most frequently used and important diagnostic factors were ‘pain pattern’ and ‘range of motion (ROM)’ (average score = 4.6 and 4.5, respectively). Other diagnostic factors that had an average score of more than 4.0 were ‘activities of daily living (ADL)’ and ‘factors associated with life (social history)’ (4.2 and 4.1, respectively) (Table 2).

In relation to ‘pattern identification’, the diagnostic tool of KM, the common types were presented as ‘*Qi and blood stagnation*’, ‘*Phlegm-dampness*’, ‘*blood stasis*’, and ‘*wind-cold-dampness*’ (75.3%, 47.7%, 39.2%, and 36%, respectively).

**Figure 1:**
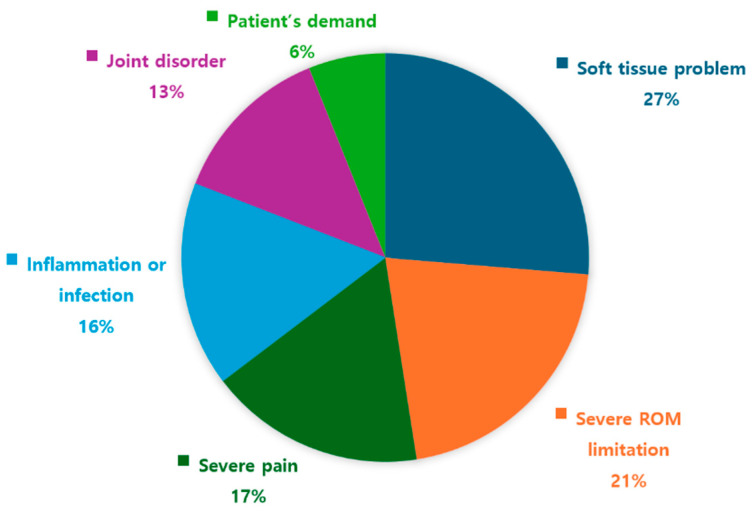
Factors indicating the need for a radiological test or laboratory test. ROM, range of motion.

For the necessity of a radiological test or laboratory test, about 85% of KMDs answered positively that they are necessary for diagnosis for shoulder pain [Yes, n = 672 (85.3%)]. The factors that determined a need for these tests were ‘the soft tissue problem including rotator cuff rupture or calcification (n = 534, 79.3%)’, ‘severe ROM limitation (n = 431, 64.0%)’, ‘severe pain (n = 348, 51.7%)’, ‘inflammation or infection (n = 329, 48.9%)’, ‘joint disorder including fracture and arthritis (n = 264, 39.2%)’, and ‘patients’ demand (n = 124, 18.4%)’ (Figure 1).

Social history included job type, the environment of working workplace, and all other factors that are unchangeably associated with daily life.

Special tests included the drop arm test, impingement test, Apley scratch test, and other tests that can be used to differentiate the reason for shoulder pain.

Responses for the frequency and importance of diagnostic factors were based on a five-point scale: never, sometimes, usually, often, and always. Those responses were calculated to the average score by assigning a numerical value to each response option.

The major factor indicating the need for a radiological rest or laboratory test was ‘soft tissue problem including rotator cuff rupture or calcification’. The other factors, including ‘severe ROM limitation’, ‘severe pain’, and ‘inflammation or infection’, presented a similar proportion between 16% and 21%. Those factors are associated with the importance of precise diagnosis.

### 3.4. Treatments

#### 3.4.1. Overall

The three most important treatment goals were ‘improvement in pain (n = 760, 32.3%)’, ‘improvement in ROM (n = 756, 32.1%)’, and ‘improvement in quality of life (QoL) (n = 715, 30.4%)’. In terms of the frequency of treatment, most responses were concentrated between two times a week and three times a week [two times, n = 331 (42.0%); three times, n = 408 (51.8%)].

The most frequently used treatment was ‘Acupuncture (n = 776, 98,5%)’. Other treatments such as ‘cupping therapy (n = 631, 80.1%)’, and ‘pharmacopuncture (n = 628, 79.7%)’ were also followed.

In terms of the importance of treatments, the overall average score was 5.1. The average scores of each of the six highest-scoring types were 6.6 for acupuncture, 6.0 for pharmacopuncture, 5.7 for cupping therapy, 5.5 for herbal medicine, 5.3 for Chuna/manual therapy, and 5.2 for physical modalities (Table 3).

Responses for the importance of treatments were based on a seven-point scale: unimportant, slightly important, somewhat important, moderately important, quite important, very important, and extremely important. The average score was calculated by assigning a numerical value to each response option and then computing the weighted average.

#### 3.4.2. Acupuncture

‘Ah-Shi point’ was most frequently used when KMDs performed acupuncture (n = 700, 88.8%). ‘Body acupuncture’ was also a major part of acupuncture (n = 389, 49.4%). ‘Sahm’s acupuncture (n = 155, 19.7%)’, ‘Dong’s acupuncture (n = 122, 15.5%)’, and ‘Five elements acupuncture (n = 81, 10.3%)’ were used by more than 10% of KMDs.

In relation of the common region for acupuncture, the major proportion of responses was presented similarly: ‘posterior and superior part of scapular spine (n = 646, 27.9%)’, ‘posterior and inferior part of scapular spine (n = 588, 25.4%)’, and ‘lateral part of shoulder region (n = 496, 21.4%)’.

#### 3.4.3. Pharmacopuncture

‘*Jungsongouhyul Yakchim* (n = 425, 53.9%)’ and ‘bee venom (n = 353, 44.8%)’ were mainly used for pharmacopuncture, and ‘*Hwangryeon Haedok Yakchim (Coptis Detoxification Herbal Acupuncture)*’ was used by more than 20% of KMDs (n = 175, 22.2%).

‘LI15 (*Jian Yu*) (n = 601,27.3%)’, ‘SJ14 (*Jian Liao*) (n = 366, 16.6%)’, and ‘GB21 (*Jian Jing*) (n = 334, 15.2%)’ were the most commonly used points for pharmacopuncture. Other points included ‘SI9 (*Jian Jing*) (n = 213, 9.7%)’, ‘SI11 (*Tian Zong*) (n = 143, 6.5%)’, and ‘SI14 (*Jain Wai Shu*) (n = 112, 5.1%)’ that were used by approximately 5% to 10% of KMDs.

#### 3.4.4. Chuna/Manual Therapy

‘Chuna therapy’ was the most frequently used manual therapy (n = 352, 44.7%), followed by ‘musculotendinous releasing manual therapy (n = 262, 33.2%)’ and ‘Daoyin therapy (n = 252, 32%)’.

Among Chuna techniques, more than 50% of KMDs used simple Chuna therapy, including ‘joint extension and mobilization (n = 282, 80.1%)’ and ‘fascia Chuna therapy (n = 230, 65.3%)’. ‘Complex Chuna therapy’ was not used as much as ‘Simple Chuna therapy’ [shoulder joint displacement correction, n = 76 (21.6%); spine displacement correction, n = 72 (20.5%)].

The ‘head and neck region (n = 269, 76.4%)’ and ‘shoulder girdle (n = 259, 73.6%)’ were the most common regions for Chuna therapy, followed by ‘glenohumeral joint (n = 103, 29.3%)’ and ‘thoracolumbar region (n = 38, 26.1%)’.

#### 3.4.5. Herbal Medicine

Various types of herbal medicines were used. ‘Oyaksunki-san (n = 267, 33.9%)’, ‘Seogyeong-tang (n = 225, 28.6%)’, ‘Gyeonbi-tang (n = 212, 26.9%)’, ‘Wujeok-san (n = 204, 25.9%)’, ‘Danggui-susan (n = 172, 21.8%)’, and ‘Gaegyeol-seogyeong-tang (n = 164, 20.8%)’ were the commonly used herbal medicines. ‘*Hoisu-san*’, ‘*Erchen-tang*’, ‘*Banxia Jianzhu Tang*’, and ‘*Seogeun-tang*’ were also used but less commonly, and 10.7% of KMDs responded ‘others’.

Regarding the duration of treatment with herbal medicine, ‘2–4 weeks (n = 383, 48.6%)’ was the most common, followed by ‘1–3 months (n = 278, 35.3%)’.

#### 3.4.6. Cooperation Between KM and Western Medicine

More than half of the KMDs acknowledged the need for cooperation between KM and Western medicine (WM). ‘Anti-inflammatory agents (n = 207, 46.7%)’, and ‘surgery (n = 199, 44.9%)’ were the commonly needed WM treatments alongside KM. When deciding the need for medical cooperation, ‘severe pain (n = 274, 61.9%)’, and ‘symptoms of inflammation or infection (n = 234, 52.8%)’ were most commonly considered.

However, the realistic percentage of performed medical cooperation was 36.1%, less than 40%.

### 3.5. Progress and Prognosis

For assessing the improvement, a majority of the KMDs suggested evaluation ‘within 1 month after first treatment (n = 522, 73.2%)’ or ‘between 1 and 3 months after first treatment (n = 141, 19.8%)’.

Regarding the importance of prognostic factors, the initial condition at first visit, including ‘ROM’ and ‘pain’, was considered the most important (4.6 and 4.6, respectively). ‘ADL’, ‘pain duration’, and ‘social history’ were also considered important (4.2, 4.2, and 4.1, respectively) (Supplementary Table S1).

### 3.6. Perception of KM in Shoulder Pain

Regarding the effectiveness of KM treatment on shoulder pain, it was agreed to have a positive impact on the prognosis of ‘improvement in pain’, ‘improvement in movements’, ‘improvement in QoL’, ‘improvement in work efficiency’, and ‘reduction in necessary of medication or surgery’ (4.7, 4.7, 4.6, and 4.5, respectively).

Regarding the safety of KM treatment, the average safety score for the 10 types of KM treatment was 4.0. Treatments with average scores greater than 4.0 were ‘acupuncture (4.7)’, ‘cupping therapy (4.5)’, ‘physical modalities (4.4)’, ‘herbal medicine (4.3)’, and ‘Daoyin therapy (4.2)’. ‘Chuna/manual therapy (4.0)’, ‘moxibustion (3.9)’, and ‘pharmacopuncture (3.8)’, which had average safety scores near 4.0 (Supplementary Table S2).

## 4. Discussion

This study is the first survey to investigate the clinical environment of shoulder pain treatment among KMDs. We found that the most common conditions of shoulder pain were frozen shoulder, sprains and strains, and rotator cuff problems. Acupuncture and pharmacopuncture were identified as the most important treatments, with a short average treatment period of 3 months and high satisfaction rates. Additionally, ADL and social history were emphasized as significant diagnostic factors. KMDs primarily used combination treatments, with Chuna therapy being the most frequently applied manual therapy.

The online survey was filled out by 788 respondents in three weeks. Considering that there are currently around 20,000 KMDs providing clinical care [2], approximately 4% of active KMDs responded to the survey. This number may not be large compared to the entire number of KMDs. However, KMDs with varying years of clinical experience responded in proportional distribution. Furthermore, the proportions related to gender and specialization subject were close to those that were investigated in an annual report on KM2. Therefore, it can be regarded as representative of the KMD population.

The number of first-visit patients with shoulder pain every month is reasonable considering that shoulder pain is among the top 10 most prevalent conditions with substantial total medical insurance costs in 2022, Korea [2,3]. The three main diseases for shoulder pain were reported to be ‘frozen shoulder,’ ‘sprain and strain,’ and ‘rotator cuff problem,’ in that order. The common age of patients with shoulder pain ranged from 40 to 60 years old. The three major diseases and the age of the patients in this study were similar to the previous reports [1,4,5,6,7,11,16].

The interesting points of our study are the average treatment period and the rate of satisfaction. More than 80% of KMDs reported the average treatment period as within 3 months. It is short considering that the average recovery time for shoulder pain mentioned in previous studies focused on frozen shoulder or rotator cuff rupture is 3 months [17]. The satisfaction score for KM treatment in this study was concentrated on 7–8 out of 10. We assume that the higher satisfaction could result from a shorter treatment period.

Regarding diagnosis, ‘pain pattern,’ ‘ROM,’ ‘ADL,’ and ‘social history’ were considered important with scores greater than 4.0. Pain aspect and ROM have been mentioned as one of the main diagnostic factors [4,5,6,7,16]; however, ADL and social history were also noted in this KM survey. This finding shows that the comprehensive diagnosis by KMDs is based not only on the disease characteristics but also on the patients’ subjective symptoms and other daily life factors, including lifestyle, occupation, continuous work, environment related to work, alignment of the body, and posture. The comprehensive diagnosis is a unique feature of KM.

In terms of treatments, we found that most KMDs used combination treatments consisting of three to five treatments together and did not rely only on a single therapy. The types of non-surgical treatments are much more diverse in KM than those in WM, which are only of two or three types [18]. The average score of the importance of KM treatments was 5.1 out of 7. Furthermore, except for two treatments for which the importance scores were lower than 4.0, the overall average score of importance was 5.5. Among the different treatments, acupuncture and pharmacopuncture were considered the most important for shoulder pain. It is reasonable that both these treatments are used most frequently and are regarded as important, considering that both have been well known to reduce pain [19,20,21,22]. Cupping therapy, herbal medicine, and Chuna/manual therapy are followed as important treatment choices. These results align with the most frequently used treatments in KM.

Regarding the details of various treatments, the major points of acupuncture and pharmacopuncture were similar. Most points focused on the painful region, the posterior part of the scapular and the lateral part of the shoulder region. The common acupoints, LI15 (*Jian Yu*), SJ14 (*Jian Liao*), GB21 (*Jian Jing*), SI9 (*Jian Jing*), SI11 (*Tian Zong*), and SI14 (*Jain Wai Shu*), are distributed in the abovementioned regions of the shoulder. With an anatomical background, these acupoints are related to insertion, origin, and muscle belly of the rotator cuff and the glenohumeral joint. These are the main sites of injection therapy for shoulder pain and are well-known sites as the reason for shoulder pain in shoulder lesions [23,24,25]. These reports indicate that KMDs’ clinical decisions are based on the pathophysiological background of shoulder pain.

Chuna therapy is a manual therapy that involves correcting the imbalance and misalignment of the body to control the neuromusculoskeletal disorder. This was the most frequently used manual therapy. Unlike other manual therapies, Chuna therapy is covered under the Korean public health insurance, and there is a well-developed standard Chuna manual for shoulder pain [16,26]. These reasons could have resulted in the high frequency of application of Chuna therapy. Other manual therapies, such as Daoyin therapy, can also be used frequently once a standard manual is developed, and if this treatment is covered by the public health insurance. Chuna therapy is not only independently applied to the shoulder region, but the neck and thoracic regions are also often involved. The rationale is that the neck and thoracic regions are key areas that affect alignment for proper physiological function of the upper body, including the shoulder girdle [16]. These reports indicate the wholistic perspective of KM.

In terms of herbal medicines, none of the herbal medicines were prescribed by more than one-third of the respondents. There was a wide variety of herbal medicines prescribed, suggesting that KMDs decide the herbal treatment after examining the patient’s characteristics and considering the pathophysiological mechanisms.

In terms of progress and prognosis, most KMDs agreed that follow-up assessments should be conducted within 1 month. This follow-up duration is shorter than for other forms of medicine [17], and it can show that the patients’ symptoms are more closely observed in KM than in other forms of medicine.

In Korea, most medical clinics are categorized as KM clinics, WM clinics, and clinics that have a combination of both. In terms of medical cooperation, most KMDs agreed that it is necessary, depending on the severity of pain and the presence of symptoms related to inflammation or infection. Although more than half of the KMDs acknowledged the need for medical cooperation, in reality, the score for medical cooperation was lower than 4 out of 10. The discrepancy could be due to more than half of the KMDs working in KM clinics without cooperation.

In this study, the three most important diagnostic factors, including the purpose of treatment, prognostic factors, and positive effects of KM treatment, were related to pain, ROM, and ADL. This notable finding indicates that KMDs are capable of providing a reasonably good diagnosis, treatment, and assessment with consistent decision-making.

Compared to previous studies [10,11,12], which reviewed other published studies or medical data from several hospitals, the results of the present study are similar in terms of the common age of patients, main diseases for shoulder pain, duration of treatment, and details of treatment including acupoints and various types of herbal medicines. And it is newly discovered how the KMDs use various treatments in clinical real-world settings, and the perception of KM for shoulder pain.

This study has some limitations. First, the sample size was small due to the short duration of the survey (3 weeks). Although we aimed to collect responses that closely reflected the demographic distribution of the overall KMD population, the final response rate was approximately 3% (788 responses). This low response rate may nonetheless limit the generalizability of the findings. Additionally, since the questionnaire did not allow free text responses, some conditions of KM treatment may not have been fully captured. Furthermore, the survey was administered only to KMDs, so the demands of patients with shoulder pain visiting KM clinics were not addressed.

Nevertheless, this is the first survey to directly assess the clinical practices of KMDs in treating shoulder pain, providing valuable data for updating the KCPG. Understanding the actual clinical practices of KMDs is essential to accurately reflect their needs and demands when updating the guidelines. While the sample size may not fully represent all KMDs, this study serves as a foundational step for future research and evidence-based clinical guidelines. The insights from this survey may serve as preliminary data to inform future updates to the KCPG for shoulder pain. These findings, when combined with systematic reviews, evidence-based assessments, and expert consensus on treatment modalities, will contribute to refining the guidelines to better reflect the current state of Korean medicine treatment for shoulder pain.

## 5. Conclusions

After surveying the KMDs to investigate the clinical environment of treatment for shoulder pain, we found that the KMDs examined and treated many patients with shoulder pain, and their comprehensive diagnosis was based on pathophysiological mechanisms and the wholistic view of KM. The KMDs considered various factors such as pain, ROM, lifestyle, and occupation when identifying the cause of shoulder pain. Furthermore, they used combination treatments comprising four–five therapies. The improvement in pain intensity, ROM, and ADL was facilitated by treating local lesions using acupuncture and pharmacopuncture, as well as alleviating individual factors that cause shoulder pain using Chuna therapy and herbal medicines. The insights obtained through this survey may help inform future updates to the KCPG for shoulder pain, particularly by contributing practice-based perspectives to the broader evidence-based guideline development process.

## Figures and Tables

**Table 1:** General characteristics of participants.

Characteristic		n (%)
Sex	Total Number	788 (100)
	Male	585 (74.2)
	Female	203 (25.8)
Clinical experience (years)	~5	100 (12.7)
	6~10	189 (24)
	11~15	159 (20.2)
	16~20	118 (15)
	20~30	178 (22.6)
	30~	44 (5.6)
Institutional working	KM clinic	554 (70.3)
	KM hospital (including university or not)	158 (20.1)
	Nursing hospital	25 (3.2)
	Public health center	22 (2.8)
	Hospital (working with Western medicine)	11 (1.4)
	Military as a medical officer	7 (0.9)
	Government organization	5 (0.6)
	National medical institutions	4 (0.5)
	Others	2 (0.3)
Academic ability	Bachelor	364 (46.2)
	Graduate school	162 (20.6)
	KM specialist	76 (9.6)
	Graduate school + KM specialist	186 (23.6)
Specialization subject	Internal Medicine	128 (18.2)
	department of acupuncture and moxibustion	122 (17.4)
	Rehabilitation Medicine	96 (13.7)
	Gynecology	32 (4.6)
	Department of Sasang Constitution	29 (4.1)
	Ophthalmology, Otorhinolaryngology, and Dermatology	21 (3.0)
	Neuropsychiatry	15 (2.1)
	Pediatrics	10 (1.4)
	None	250 (35.6)

KM, Korean medicine.

**Table 2:** Frequency and importance of diagnostic factors using a five-point scale.

Diagnostic Factors	Score of Frequency and Importance
Pain pattern	4.6
ROM	4.5
ADL	4.2
Social history	4.1
Alignment of neck and shoulder	3.7
Special test	3.5
Past history	3.4
Pattern identification	3.1
Radiological test	3.0
Laboratory test	1.9

ROM, range of motion; ADL, activities of daily living.

**Table 3:** Importance of treatment using a seven-point scale.

Treatments	Score of Importance
Acupuncture	6.6
Pharmacopuncture	6.0
Cupping therapy	5.7
Herbal medicine	5.5
Chuna/manual therapy	5.3
physical modalities	5.2
Daoyin therapy	5.0
Moxibustion	4.5
Acupotomy	4.0
Thread embedding acupuncture	3.6
Overall average	5.1

## Data Availability

The datasets generated and/or analyzed during this study are available from the corresponding author on reasonable request.

## Supplementary Materials

The following supporting information can be downloaded at: https://www.mdpi.com/article/10.3390/healthcare13131482/s1. Table S1. Importance of prognostic factors; Table S2. Safety of KM treatments.
