# Supplementary material for: Survey of Korean Medicine Doctors’ Practices in Treating Shoulder Pain: A Cross-Sectional Study
Source: Healthcare (Basel). 2025 Jun 20;13(13):1482. doi: 10.3390/healthcare13131482 (PMC12248967; doi:10.3390/healthcare13131482)
Supplement: Supplementary file 1 [file healthcare-13-01482-s001.zip › healthcare-3574726-supplementary.pdf]

Supplementary Materials

Supplementary Table S1 Importance of prognostic factors

Prognostic factors	Score of importance
ROM at first visit	4.6
Pain at first visit	4.6
ADL	4.2
Pain duration	4.2
Social history*	4.1
Causative disease	3.9
Alignment of neck and shoulder	3.7
Radiological test	3.5
Past history	3.4
Pattern identification	3.3
Korean-Western medical cooperation	3.1

ROM; Range of motion, ADL; activities of daily living

* Social history included the job, environment of working place, and all other factors that are unchangeably associated with daily life.

Responses for the importance of prognostic factors were based on five-point scale: not important, less important, important, more important, and most important. The average score was calculated as follows:

$$\frac{[(\text{not important})\text{x1} + (\text{less important})\text{x2} + (\text{important})\text{x3} + (\text{more important})\text{x4} + (\text{most important})\text{x5}]}{\text{Total number of responses}}$$

Supplementary Table S2 Safety of KM treatments

Treatment	Score of safety
Acupuncture	4.7
Cupping therapy	4.5
Physiotherapy	4.4
Herbal medicine	4.3
Daoyin therapy	4.2
Chuna/manual therapy	4.0
Moxibustion	3.9
Pharmacopuncture	3.8
Thread embedding therapy	3.1
Acupotomy	3.0
Average	4.0

Responses for the safety of KM treatment were based on a five-point scale: very low, low, usual, high, and very high. The average score was calculated as follow:

$$\frac{[(\text{very low})\text{x1} + (\text{low})\text{x2} + (\text{usual})\text{x3} + (\text{high})\text{x4} + (\text{very high})\text{x5}]}{\text{Total number of responses}}$$
